# Radiological Assessment of Inter- and Intra-observer Reliability in Hip Migration Measurements in Children With Cerebral Palsy at a Tertiary Referral Center

**DOI:** 10.7759/cureus.98870

**Published:** 2025-12-10

**Authors:** Joseph Sajeev, Binu Kurian, Jaya V Lal, Arun George, Joe Joseph

**Affiliations:** 1 Orthopedics, St. John’s Medical College Hospital, Bengaluru, IND; 2 Radiology, St. John’s Medical College Hospital, Bengaluru, IND

**Keywords:** acetabular dysplasia, acetabular index, cerebral palsy (cp), hip migration index, hip surveillance

## Abstract

Background

Hip migration is a common deformity in children with cerebral palsy (CP). Although children with CP often have anatomically normal hips at birth, progressive spasticity can lead to hip subluxation, dislocation, and acetabular dysplasia over time. Early recognition of hip migration is crucial because timely intervention can significantly change the prognosis and reduce morbidity. The aim of this study was to evaluate the inter- and intra-observer reliability of radiographic hip migration measurements in children with CP.

Materials and methods

We conducted a prospective observational study from 2020 to 2022 to determine inter- and intra-observer variation in the diagnosis of hip migration among children with CP. Eligible participants were recruited from the clinic and followed up for a period of two years. They underwent serial supine AP pelvic radiographs at six-month intervals. The radiographic parameters evaluated were Reimer’s migration percentage (MP) and the acetabular index (AI). Four observers with varying levels of clinical experience independently assessed the radiographs. Inter- and intra-observer agreement was analyzed using intraclass correlation coefficients (ICCs) and standard statistical methods.

Results

Eighteen children (mean age 12.2 years, range 2-18 years, SD 7.72) with spastic CP were included in the study. To ensure consistency and avoid inter-hip variability in the same patient, the hip showing greater displacement served as the unit of analysis. Based on the Gross Motor Function Classification System, nine were classified as level IV, seven as level III, and two as level V. The MP demonstrated consistently high inter- and intra-observer reliability, with ICCs and 95% CIs of 0.999 (0.988-1.000) at baseline and 0.999 (0.999-1.000) at endline. Similarly, the AI also showed excellent inter- and intra-observer reliability, with ICCs and 95% CIs of 0.992 (0.984-0.997) at baseline and 0.998 (0.997-0.999) at endline. These findings indicate strong reproducibility for both measurements across observers and time points. MP analysis showed a significant increase from baseline to endline (Wilcoxon Z = -3.724, p = 0.0002), indicating progression of hip migration. AI also increased significantly over time (paired t = -3.944, p = 0.0010), reflecting the progression of acetabular dysplasia. Both represent secondary outcomes of the study.

Conclusions

This study demonstrates that both MP and AI provide comparable and clinically acceptable levels of inter- and intra-observer reliability, with a slight advantage for MP. These results underscore the value of MP as a primary surveillance metric while reaffirming AI as a reliable adjunct for evaluating acetabular morphology in children with CP. By contributing additional evidence on the reproducibility of these measurements, our study supports their continued use within structured hip surveillance programs. Further multicenter, prospective studies are needed to validate these findings in broader populations and to strengthen the foundation for standardized, universally applicable surveillance protocols.

## Introduction

Cerebral palsy (CP) comprises a group of nonprogressive neurological disorders that impair mobility and postural control. With advances in neonatal and pediatric care, survival among infants and children with CP has increased worldwide, making it the most common physical impairment in this population [1].

Among musculoskeletal deformities associated with CP, hip migration is the second most common after spastic equinus of the foot [1]. Although the hips are typically anatomically normal at birth, progressive spasticity, impaired proprioception, and abnormal postural alignment contribute to gradual displacement of the hip joint, often leading to dislocation and acetabular dysplasia over time [2]. Aberrant muscle forces, altered joint reaction forces, and changes in force vector orientations further contribute to acetabular deformity.

Hip surveillance programs, which incorporate routine supine AP pelvic radiographs from early childhood through skeletal maturity, have proved effective in identifying CP-related hip dysplasia in its early stages and facilitating timely intervention [3]. These programs are increasingly being integrated into routine care at regional and national levels through collaboration with multidisciplinary teams [4].

The purpose of hip surveillance programs is the early detection of progressive hip displacement, enabling timely orthopedic referral and intervention [5]. Accurate radiographic assessment is essential to these programs. Reimer’s migration percentage (MP) remains the gold standard metric for quantifying hip displacement, while the acetabular index (AI) provides complementary information on acetabular morphology and dysplasia [6]. Together, these parameters support risk stratification and guide clinical decision-making for the early diagnosis of progressive hip displacement in children with CP.

## Materials and methods

We conducted a prospective observational study between 2020 and 2022 to assess inter- and intra-observer variability in the radiographic diagnosis of hip migration among children with CP. A minimum follow-up period of two years was maintained for all participants. A major limitation was the difficulty in ensuring consistent follow-up during the COVID-19 pandemic, which affected patient attendance and completeness of data collection. Additionally, as this was a single-center study, the findings may have limited external validity and cannot be readily generalized to broader populations.

Study population

Children aged 2 to 18 years with spastic CP classified as levels III-V according to the Gross Motor Function Classification System (GMFCS) [7] were eligible for inclusion. Children with congenital hip or spinal disorders, prior surgical intervention, or significant medical comorbidities were excluded from the study. A total of 18 patients were enrolled, based on an a priori sample-size estimation for an expected ICC of 0.93 for MP and AI measurements with 10% precision at a 95% confidence level. Written informed consent was obtained from their parents or legal guardians, and ethical approval for the study was secured from the institutional review board before commencement (IEC study no. 428/2020).

Unit of analysis

CP commonly results in bilateral hip involvement, often with asymmetry in the severity of migration. To ensure consistency and avoid inter-hip variability within the same patient, the hip demonstrating the greater degree of displacement on clinical and radiographic assessment was designated as the unit of analysis for this study. In all enrolled children, the left hip consistently exhibited more severe migration compared with the right. Therefore, although most participants had bilateral hip involvement, radiographic measurements and subsequent analyses were standardized using the more affected left hip. This methodological approach enhanced uniformity in data interpretation and improved the reliability of comparative evaluations across the study.

Standardized radiographic assessment

Standardized supine AP pelvic radiographs were obtained at six-month intervals for all participants. In cases of bilateral hip involvement, the most severely affected hip was used as the reference for assessment. Radiographs were acquired with the child positioned supine, legs kept parallel, and patellae facing upward. Spinal support was provided to minimize lumbar lordosis caused by hip flexion contractures [8]. Radiology staff were instructed to ensure neutral pelvic obliquity and reduce lumbar lordosis by gently elevating the legs. When children were unable to lie completely flat due to persistent hip flexion contractures, a knee bolster was placed to further minimize pelvic tilt.

Visualization of the triradiate cartilage is essential for accurate measurement of the MP; therefore, anterior or posterior pelvic tilt was corrected by adjusting leg elevation as needed. Parental involvement formed an important component of the surveillance protocol, including providing consent for imaging, ensuring adherence to follow-up intervals, and agreeing to orthopedic referral when required [9].

Observer training

Before initiating the radiographic assessments, all four observers developed a consensus-based written protocol to standardize the measurement technique. The observer team consisted of a radiology consultant with 25 years of experience (Observer 1), an orthopedic consultant with 25 years of experience (Observer 2), and two first-year residents: one from orthopedics (Observer 3) and one from radiodiagnosis (Observer 4).

A structured one-month training period was conducted, during which the observers met for one hour each evening. The radiology consultant provided focused instruction on reviewing pelvic radiographs using the picture archiving and communication system (PACS) and demonstrated the correct placement of each measurement line. These sessions were intended to reduce inter-observer variability and ensure uniform application of the protocol.

The radiographic parameters assessed included Hilgenreiner’s line (a horizontal line passing through both triradiate cartilages), Perkin’s line (a vertical line drawn perpendicular to Hilgenreiner’s line at the superolateral margin of the acetabulum), Reimer’s MP, calculated as the percentage of the femoral head lying lateral to Perkin’s line, and the AI [10], defined as the angle between a horizontal line through the triradiate cartilages and a line along the acetabular roof.

Statistical analysis

Data analysis was performed using IBM SPSS Statistics for Windows, Version 25.0 (Released 2017; IBM Corp., Armonk, NY, USA). Descriptive statistics were expressed as means and standard deviations for normally distributed continuous variables (AI) and as medians with IQRs for non-normally distributed variables (MP). Categorical variables were summarized using frequencies and percentages. Inter-rater reliability was assessed using the intraclass correlation coefficient (ICC) with a 95% CI, applying a two-way mixed-effects model (where subject effects are random and measurement effects are fixed) with a consistency definition to evaluate variability in radiographic measurements of hip migration among children with CP. The Wilcoxon signed-rank test was applied to compare baseline and endpoint values for non-normally distributed variables such as Reimer’s MP, while paired t-tests were used to compare baseline and endpoint values for normally distributed variables such as the AI.

## Results

Eighteen children with spastic CP were included in the study, with a mean age of 12.2 years (range: 2-18 years; SD: 7.72). According to the GMFCS, nine children were classified at level IV, seven at level III, and two at level V (Table 1).

**Table 1:** Descriptive statistics GMFCS, Gross Motor Function Classification System; L, left; MP, migration percentage; R, right

Parameter	Frequency
Mean age (years)	12.2
Gender (M:F)	13:05
GMFCS	
GMFCS III	7
GMFCS IV	9
GMFCS V	2
Laterality (R:L)	0:18
MP range	
0-10	7
10-20	6
20-30	3
>30	2

Table 2 presents the comparison of baseline MP (BMP) and end MP among the observers.

**Table 2:** Comparison of BMP and EMP among observers BMP, baseline migration percentage; EMP, end migration percentage

Observer	BMP (median (range))	EMP (median (range))
Observer 1	14.2 (9.4-22.4)	20.7 (17.3-27.2)
Observer 2	14.2 (10.2-22.5)	23.3 (17.5-29.2)
Observer 3	14.5 (9.2-22.4)	20.9 (17.5-26.9)
Observer 4	14.6 (9.3-23.1)	20.8 (17.3-20.8)

For MP, the BMP medians (IQR) for the four observers were 14.2 (9.4-22.4), 14.2 (10.2-22.5), 14.6 (9.3-22.4), and 14.6 (9.3-23.1), while the endpoint MP medians were 20.7 (17.3-27.2), 23.4 (17.5-29.2), 20.9 (17.5-26.9), and 20.9 (17.3-20.8).

Table 3 shows the baseline AI (BAI) and endline AI (EAI) measurements among the four observers.

**Table 3:** Comparison of BAI and EAI among observers BAI, baseline acetabular index; EAI, endline acetabular index

Observer	BAI (mean ± SD)	EAI (mean ± SD)
Observer 1	16.7 (4.40)	21.2 (7.21)
Observer 2	16.4 (4.46)	21.0 (7.30)
Observer 3	16.7 (4.66)	21.1 (7.34)
Observer 4	16.5 (4.42)	21.1 (7.27)

For AI, the baseline mean (standard deviation) values for the four observers were 16.7 (4.40), 16.4 (4.46), 16.7 (4.66), and 16.5 (4.42), while the EAI values were 21.2 (7.21), 21.0 (7.30), 21.1 (7.34), and 21.1 (7.27).

Statistical analysis demonstrated consistently high inter- and intra-observer reliability across all four evaluators for MP. The BMP ICC, calculated using a two-way mixed-effects model with a consistency definition, was 0.999 (95% CI: 0.999-1.000; p < 0.001). The endline MP ICC was 1.000 (95% CI: 1.000-1.000; p < 0.001). Similarly, the BAI ICC was 0.998 (95% CI: 0.996-0.999; p < 0.001), and the EAI ICC was 1.000 (95% CI: 0.999-1.000; p < 0.001) (Table 4).

**Table 4:** ICCs for MP and AI AI, acetabular index; ICC, intraclass correlation coefficient; MP, migration percentage

Variable	Individual variable	ICC	95% CI	
			Lower bound	Upper bound
MP	Baseline	0.999	0.998	1.000
	Endline	0.999	0.999	1.000
AI	Baseline	0.992	0.984	0.997
	Endline	0.998	0.997	0.999

**Figure 1:**
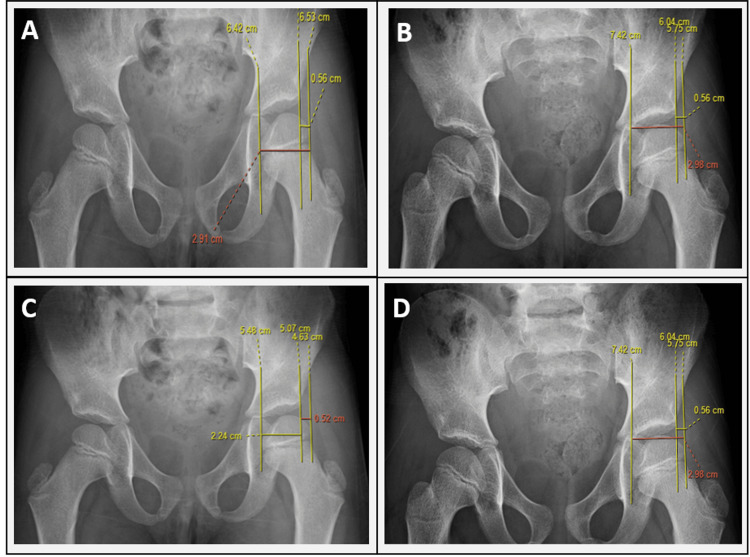
MP as measured by different observers A = Observer 1; B = Observer 2; C = Observer 3; D = Observer 4 MP, migration percentage

Pelvic radiographs were evaluated using PACS software. The comparison of MP measurements among the observers is illustrated in Figure 1.

Progression analysis for MP was performed per hip, with values measured by the radiodiagnosis consultant. The Wilcoxon signed-rank test demonstrated a Z value of -3.724 (p = 0.0002), indicating a statistically significant increase in MP over time. This finding reflects progressive worsening of hip migration and represents a secondary outcome of the study.

Similarly, for AI, measurements per hip obtained by the radiodiagnosis consultant were compared between baseline and endline using a paired t-test. The analysis demonstrated a t value of -3.944 (p = 0.0010), confirming a significant increase in AI over time. This upward trend in AI also reflects the progression of the underlying hip pathology and is considered a secondary conclusion of the study.

**Figure 2:**
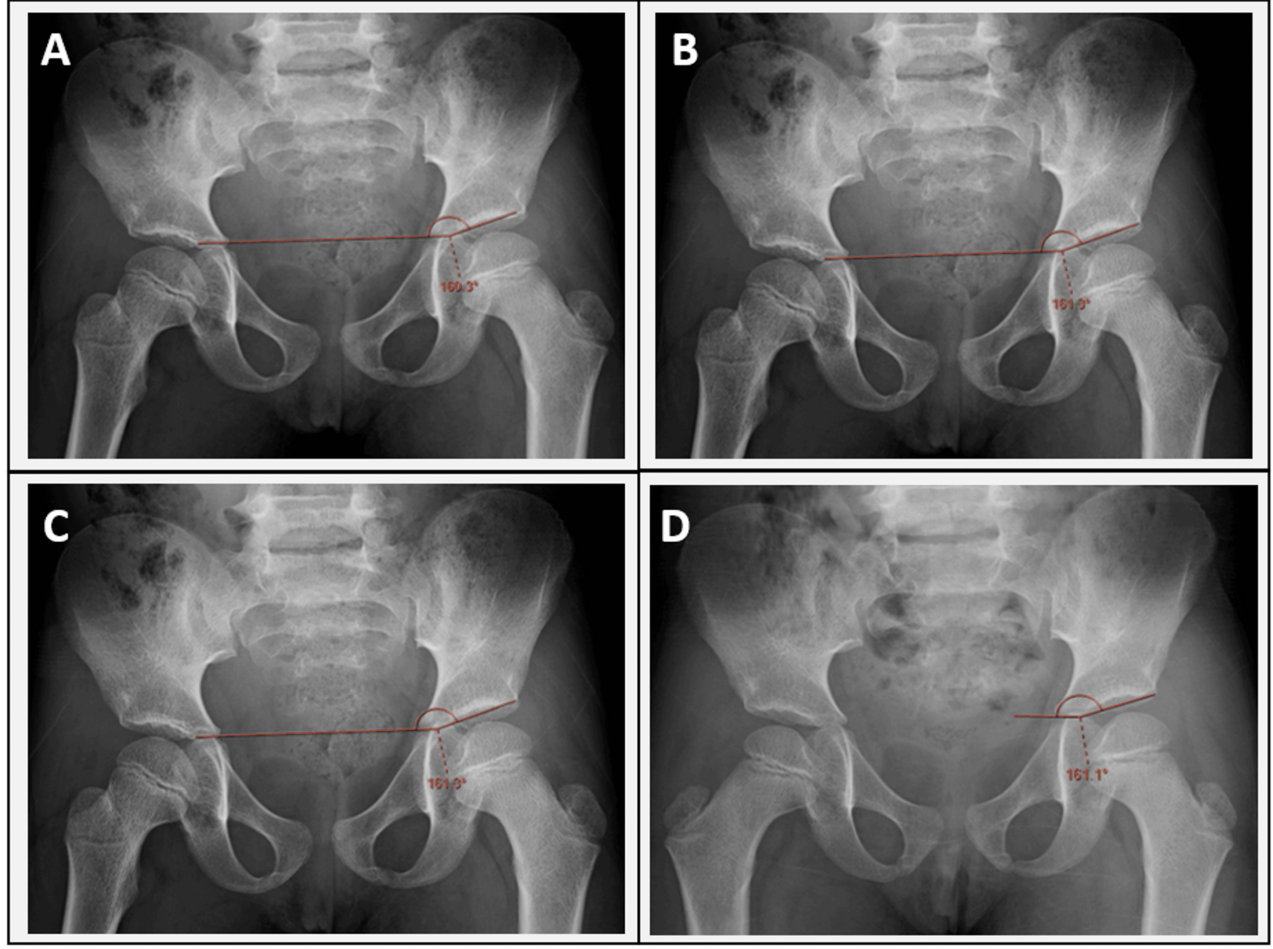
AI measured by different observers A = Observer 1; B = Observer 2; C = Observer 3; D = Observer 4 AI, acetabular index

Pelvic radiographs used for AI measurements and evaluated using PACS software are presented in Figure 2.

## Discussion

An efficient and standardized surveillance strategy is essential for children with spastic CP to monitor hip displacement or dislocation and to facilitate early detection, which can significantly improve long-term quality of life. The aim of the present study was to establish consensus among observers with varying levels of expertise regarding the reliability of two key radiographic parameters: Reimer’s MP and the AI. Consistent application of these measures, even in primary care settings with only plain radiography, can support timely detection and referral, thereby potentially reducing the need for more extensive surgical procedures later in life [11].

The most common age for initial hip displacement, defined by an MP of 33-40%, is between three and four years, with some children demonstrating displacement as early as two years of age [12]. According to Toovey et al. [13], hips are considered at risk for subluxation and dislocation when the MP is between 30% and 33%, whereas values exceeding 40% typically necessitate surgical intervention to prevent further progression. Recent evidence has further refined these thresholds. Arenas-Díaz et al. [14] proposed optimized cutoffs, reinforcing the clinical use of ≥30-33% to designate “at-risk” hips and >40% as indicative of surgical need.

Our findings demonstrated slightly higher inter- and intra-observer reliability for MP compared with AI, aligning with recent literature emphasizing the strong reproducibility of MP and its role as the most dependable parameter in hip surveillance. These observations are consistent with the conclusions of Shore et al. [15], who reported that serial MP measurement on plain radiographs remains the most practical, reliable, and clinically applicable method for hip surveillance. Kim et al. [2] demonstrated intra-rater reliability with a standard error of measurement of 8.3% for MP, highlighting the need for precise protocols to minimize variability.

In our study, MP demonstrated slightly higher inter- and intra-observer reliability than AI. This result is consistent with the Australian Hip Surveillance Guidelines [16], which, after a decade of implementation, continue to recommend MP as the gold standard for monitoring hips. A recent systematic review of European hip surveillance programs [17] also demonstrated that structured programs significantly reduced the prevalence of late hip dislocation associated with CP, highlighting the clinical value of reproducible MP measurements.

Our findings are also consistent with those of Larnert et al. [18], who reported a correlation between GMFCS level and the severity of hip migration. They found that among children classified as GMFCS level V, by the age of three years, 23% had an MP of 33%, and 17% had an MP of 40%. Similarly, in our cohort, severe hip migration was observed in 50% of children with GMFCS level IV and 11% of those with GMFCS level V. These results support the observation that abnormal muscle forces associated with higher GMFCS levels predispose children to earlier and more severe hip displacement than those at lower levels. Recent multicenter epidemiological studies further support this relationship, confirming that GMFCS level is the best predictor of hip displacement [19].

Beyond radiographic accuracy, the association of integrated hip surveillance pathways with improvements in pain, function, and quality of life underscores the broader clinical implications of reliable MP assessment [20]. This suggests that the high reproducibility of MP observed in our cohort could translate into meaningful functional benefits through earlier referral and less invasive intervention strategies.

Limitations

This study has several limitations. The heterogeneity in observer experience, including differences in seniority and areas of expertise, may have influenced the reliability estimates. Because CP typically affects both hips, the more severely affected hip was used as the unit of analysis, which may limit the interpretation of bilateral variation. The sample size was relatively small due to strict inclusion criteria aimed at creating a homogeneous cohort, which may reduce the precision of the estimates. Although standardized recommendations for obtaining AP pelvic radiographs in CP surveillance were followed, adherence could not be verified in all cases. Many children presented with significant spinal deformities and lower-extremity contractures that limited optimal positioning. In addition, radiographic acquisition could not always be standardized due to variability among radiology technicians. Follow-up was also a substantial challenge during the COVID-19 pandemic, affecting patient attendance and the completeness of data collection. As this was a single-center study, the findings may have limited external validity and may not be readily generalizable to broader populations. Finally, despite efforts to ensure methodological transparency, some aspects of the statistical reporting remain incomplete, limiting interpretability and reproducibility. Future studies should incorporate more detailed statistical reporting to align with best-practice guidelines and strengthen methodological rigor.

## Conclusions

Both MP and AI demonstrated comparable and clinically acceptable inter- and intra-observer reliability, with a slight advantage observed for MP. These results support the continued use of MP as a primary surveillance metric while reaffirming AI as a reliable adjunct for assessing acetabular morphology in children with CP. By adding evidence on the reproducibility of these measurements, this study supports their ongoing application within structured hip surveillance programs. However, multicenter prospective studies are needed to validate these findings in larger and more diverse populations and to further strengthen the foundation for standardized, universally applicable surveillance protocols.

## Appendices

Written protocol for the observers for calculations of Reimer’s migration percentage and acetabular index

Instructions to the radiology technician:

(1) Neutral adduction or abduction of the legs to correct any pelvic obliquity

(2) Patella facing upward

(3) To correct the exaggerated lumbar lordosis by elevating the legs

(4) To keep a bolster below the knees to reduce the pelvic tilt if they cannot lie flat due to hip flexion contracture

Importance of proper positioning of the patient:

(1) For accurate measurement of the migration percentage and acetabular index and for comparison of sequential radiographs

(2) MP and AI are affected by the degree of adduction or abduction of the leg

(3) Measurement of the MP requires that the triradiate cartilages be visible, and therefore, anterior and posterior pelvic tilt must be corrected

Instructions to the observers:

All observers must complete a training session that includes:

Review of anatomical landmarks

Demonstration of measurement steps on at least five sample radiographs (varied ages and severities)

Practice measuring and group discussion of discrepancies.

(1) DICOM viewer or PACS workstation with measurement tools (line, angle, and distance)

(2) Calibration tool: to use PACS calibration with a known marker or scale

(3) Data collection sheet or electronic spreadsheet to record measurements, patient ID, laterality, date, observer, and comments

Measurement of Migration Percentage - Protocol to be followed by the observers:

(1) MP represents the portion of the ossified femoral head that is not covered by the ossified acetabular roof.

(2) Draw Hilgenreiner's line (H), a horizontal line between the superior aspect of the triradiate cartilages. After attaining maturity, the most useful horizontal line is through the most inferior points of the acetabular teardrops.

(3) Draw Perkins’ line (P), perpendicular to Hilgenreiner’s line, at the lateral edge of the acetabulum.

(4) Draw lines, parallel to Perkins’ line, along the medial and lateral sides of the ossified femoral head.

(5) Measure the distance between the medial and lateral sides of the femoral head (B).

(6) Measure the distance between Perkins’ line and the lateral side of the femoral head (A).

(7) Calculate MP (A/B x 100%).

Measurement of Acetabular Index (AI) - Protocol to be followed by observers:

Using Hilgenreiner’s line as the horizontal reference, draw a second line along the acetabular roof: from the lateral edge of the acetabular sourcil (weight-bearing roof) to the triradiate cartilage (or best-fit line along the acetabular sourcil margin).

Measure the angle between Hilgenreiner’s line and the acetabular roof line - this is the AI.

Record AI in degrees to one decimal place.
